# Astrocytic Nrf2 expression protects spinal cord from oxidative stress following spinal cord injury in a male mouse model

**DOI:** 10.1186/s12974-022-02491-1

**Published:** 2022-06-06

**Authors:** Weiyi Zhao, Natalie Gasterich, Tim Clarner, Clara Voelz, Victoria Behrens, Cordian Beyer, Athanassios Fragoulis, Adib Zendedel

**Affiliations:** 1 Institute of Neuroanatomy, Uniklinik RWTH Aachen, Aachen, Germany; 2 Department of Anatomy and Cell Biology, Uniklinik RWTH Aachen, Aachen, Germany

**Keywords:** Spinal cord injury, SCI, Keap1, Nrf2, Astrocytes

## Abstract

**Background:**

Spinal cord injury (SCI) induces a multitude of deleterious processes, including neuroinflammation and oxidative stress (OS) which contributed to neuronal damage and demyelination. Recent studies have suggested that increased formation of reactive oxygen species (ROS) and the consequent OS are critical events associated with SCI. However, there is still little information regarding the impact of these events on SCI. Astrocytes are key regulators of oxidative homeostasis in the CNS and astrocytic antioxidant responses promote the clearance of oxidants produced by neurons. Therefore, dysregulation of astrocyte physiology might largely contribute to oxidative damage. Nuclear factor erythroid 2-related factor 2 (Nrf2) is the main transcriptional regulator of cellular anti-oxidative stress responses.

**Methods:**

In the current study, we hypothesized that astrocytic activation of Nrf2 protects the spinal cord post injury via suppression of neuroinflammation. Thus, using mice line with a GFAP-specific kelch-like ECH-associated protein 1 (Keap1)-deletion, we induced a hyperactivation of Nrf2 in astrocytes and further its effects on SCI outcomes. SCI-induction was performed in mice using the Infinite Horizon Spinal Cord Impactor with a force of 60 kdyn. To assess the quantitative pattern of Nrf2/ARE-activation, we included transgenic ARE-Luc mice. Data were analyzed with GraphPad Prism 8 (GraphPad Software Inc., San Diego, CA, USA). Brown–Forsythe test was performed to test for equal variances and normal distribution was tested with Shapiro–Wilk.

**Results:**

In ARE-Luc mice, a significant induction of luciferase-activity was observed as early as 1 day post-injury, indicating a functional role of Nrf2-activity at the epicenter of SCI. Furthermore, SCI induced loss of neurons and oligodendrocytes, demyelination and inflammation in wild type mice. The loss of myelin and oligodendrocytes was clearly reduced in Keap1 KO mice. In addition, Keap-1 KO mice showed a significantly better locomotor function and lower neuroinflammation responses compared to wild type mice.

**Conclusions:**

In summary, our in vivo bioluminescence data showed Nrf2-ARE activation during primary phase of SCI. Furthermore, we found that cell specific hyperactivation of Nrf2 was sufficient to protect the spinal cord against injury which indicate a promising therapeutic approach for SCI-treatment.

**Supplementary Information:**

The online version contains supplementary material available at 10.1186/s12974-022-02491-1.

## Background

Spinal cord injury (SCI) characteristically causes life-long neurological consequences such as paralysis, loss of sensation and voluntary motor function downwards the site of injury which remains a major cause of permanent disability [1]. SCI is divided into primary and secondary injuries in a pathophysiological classification; primary injury refers to the initial physical damage of the spinal cord (SC) caused by an indirect or direct external force, while the secondary injury is described by a series of physiological and pathological changes including neuroinflammation, neuronal apoptosis and excessive occurrence of oxidants, such as reactive oxygen species (ROS) [2]. However, oxidants are not necessarily harmful per se but do also play important roles at low exposition rates during redox-related signaling cascades by which they regulate and participate in physiological processes (thoroughly reviewed by Sies and Jones in 2020 [3]). The redox homeostasis under these circumstances should be understood as a range rather than a clearly defined intracellular state in which the ratio of oxidants and antioxidants within the cell is in dynamic equilibrium with a pending electron flow, a condition that is called oxidative eustress [4–6]. Nevertheless, once endogenous antioxidants are overwhelmed by an increased occurrence of oxidants, exceeding the limits of homeostatic conditions, a harmful imbalance between products of oxidation and antioxidant defenses results, known as oxidative distress (OS) [7]. Besides, oxidative damage to proteins and DNA, these oxidants attack polyunsaturated fatty acids in the membrane lipids, change membrane potentials and eventually rupture membranes leading to the release of cell and organelle contents [8]. Neurons are particularly prone to oxidative and electrophilic distress due to many factors, including a high content of polyunsaturated fatty acids, high rate of oxidative metabolic activity, intense production of reactive oxygen metabolites and relatively low antioxidant capacity [9]. Although increased OS is an important hallmark of the secondary phase of SCI [10], there is still little information about the impact of this biochemical changes associated with SCI outcome.

Astrocytes are the most abundant glial cells in the central nervous system (CNS) and among the first responders to SCI [11]. After SCI, local environment undergoes profound biochemical and cellular changes that affect astrocytes to become reactive and include astrogliosis [12]. Reactive astrogliosis is characterized by the proliferation and hypertrophy of astrocytes, which eventually leads to scar formation in epicenter of injury post SCI [13]. Astrocytes play critical roles in the normal SC; they closely interact with neurons to provide structural, metabolic and trophic support and actively participate in the modulation of neuronal excitability and neurotransmission [14]. In the presence of astrocytes, neurons are more resistant to OS induced by several compounds [15]. Astrocytes carry out this function mainly through transcription factor nuclear factor erythroid 2-related factor 2 (Nrf2)-driven genes, such as quinone oxidoreductase 1 (NQO1) and heme oxygenase-1 (HO-1) which are both preferentially activated in astrocytes but lesser in neurons [16]. Therefore, functional alterations in astrocytes (such as aggravation of OS) can shape and alter their interaction with neurons.

Nrf2, belonging to the cap ‘n’ collar subfamily of basic region–leucine zipper transcription factors, is the main transcription factor involved in the regulatory cascades during oxidative eustress responsible for the regulation of a whole battery of cytoprotective and antioxidative proteins by binding to antioxidant response elements (ARE) in their promoter region [17, 18]. The activity of Nrf2 is negatively regulated by the cytosolic regulatory protein kelch-like ECH-associated protein 1 (Keap1) [19]. Once, cells are exposed to OS or chemopreventive factors, Nrf2 is dissociated from Keap1, translocated into the nucleus, and induces the expression and up-regulation of downstream cytoprotective enzymes that attenuate tissue injury [20]. Since, Nrf2 is rapidly degraded through interactions with Keap1, increased Nrf2 expression does not necessary lead to increased Nrf2 activity [21]. Nrf2-inducers such as pharmacological activation of the Nrf2/ARE system is a pathway which leads to increased Nrf2-signaling. For example, treatment with dimethyl fumarate (DMF) results in beneficial effects in different CNS diseases, such as multiple sclerosis [22] and SCI [23]. Although the mechanism of action is not completely understood, it has been shown that dimethyl fumarate (DMF) induces Nrf2-activity by disruption of the Nrf2-Keap1 interaction due to a modification of the Keap1 protein [24]. However, all known Nrf2-inducers not only enhance Nrf2-activity but also exert various side effects within the brain and other peripheral tissues [25]. Therefore, genetic and cell type-specific targeting of Keap1 and Nrf2 represents the most suitable method to investigate Nrf2-specific effects. In the current study using *GFAP-Cre::keap1*^*flox/flox*^ KO mouse, we induced hyper-activation of the Nrf2/ARE system in astrocytes in a SCI model and we hypothesized that, astrocytic Nrf2 activation represents an important defense to limit the secondary tissue damage in the traumatized spinal cord by inhibiting the inflammation, and attenuation of demyelination.

## Methods

### Animals

All experimental procedures and animal care were approved by the review board for the care of animal subjects of the district government (LANUV, Germany) and are reported in accordance with the ARRIVE guidelines. The mice were housed and handled in accordance with the guidelines of the Federation for European Laboratory Animal Science Associations (FELASA) under standard laboratory conditions.

In this study, 10-week-old mice with following strains were included: (1) C57BL/6 J ARE luciferase reporter gene mice (Cgene, Oslo) [26] to visualize Nrf2-activation in epicenter of SC (7 days); (2) *GFAP-Cre::keap1*^*flox/flox*^ KO (in this manuscript is referred to as Keap1-KO) mice were generated by cross-breeding of GFAP-Cre expressing mice with Keap1^loxP/loxP^ mice as described in detail before [27] and (3) C57BL/6 J wild-type mice.

### Spinal cord injury

General anesthesia was initiated with isoflurane (2–3 vol. %) in an anesthetic chamber. During surgery, isoflurane (1.5–2 vol. %) was further administered via a face mask. Intraoperative analgesia was attained through injection of buprenorphine (0.05–1 mg/kg sc.) 30 min preoperatively. After the exposure of the spinal column (Th7–Th10), a laminectomy of Th8 was performed (Additional file 1: Fig. S1A). A standardized injury of the SC at this level was induced by contusion (Infinite Horizons Spinal Cord Impactor) with a force of 60 kdyn [28]. During the surgery, body temperature was maintained at 36–37 °C. Postoperative aftercare included rehydration (0.5 mL NaCl), postoperative analgesia and manual emptying of bladder twice a day until spontaneous urination returned. In the control group (sham), laminectomy without contusion of the SC was carried out to preclude possible falsifications of the results caused by the mere surgical procedure.

### ARE activity

ARE-activity measurement were performed as previously described [27]. Briefly, after shaving the back of mice, luciferin solution was prepared and used at a final concentration of 20 mg/mL. Totally, 0.2 mL luciferin solution was injected intraperitoneal. After injection, mice were anesthetized by inhaling 4% (v/v) isoflurane at a flow rate of 2 L/min oxygen for approximately 1 min and then kept at 1.5% (v/v) and 1 L/min using a mouse face mask. To ensure an adequate distribution of Luciferin in the whole body, the measurements were conducted 15 min after the injection. For each measurement, one sham and one SCI mouse was imaged simultaneously for 5 min at high-resolution settings (binning: 2, f/stop: 1) with a field of view of 7.5 cm. Luminescence signals were detected with an IVIS Lumina 100 Series Imaging device (Xenogen Corporation, vAlameda, CA), digitized and evaluated using the Living Image software 3.2. The signal intensities are expressed as average radiance (p/s/cm^2^/sr) and visualized with the help of a false color scale. Spinal cord-specific Nrf2/ARE activity was determined by correcting luminescence signals from pre-defined region of interest (ROIs) using background ROI (Additional file 1: Fig. S1B).

### Functional activity

The locomotion deficits of mice after SCI were scored in an open field according to Basso, Beattie, and Bresnahan (BBB) locomotion, where hindlimb function could be assessed (Basso et al. 1995). This is a 22-point scale that systematically details hindlimb function of joint movements, stepping ability, the degree of fine control of coordinated stepping and trunk stability. Test sessions were 4 min in duration and mice were tested every day, from days 1 to 7 post-injury and were scored by two experienced raters which were blinded to experimental groups.

### Tissue preparation

To obtain samples for molecular biology and biochemical examinations, mice were deeply anesthetized and transcardially perfused with ice cold phosphate-buffered saline (PBS), the whole SC was quickly removed, and the epicenter part was dissected, snap-frozen in liquid nitrogen, and stored at − 80 °C until further experiments. For immunohistochemistry, mice were transcardially perfused with ice cold PBS followed by a 3.7% paraformaldehyde solution (PFA, pH 7.4). For decalcification, spinal columns were incubated in 20% ethylenediaminetetraacetic acid (EDTA) for 48 h at 37 °C prior to paraffin embedding. To this end, tissue specimens were embedded in paraffin (Merck, Germany) and 5 µm paraffin sections were cut.

### Gene expression studies

For RNA isolation, tissues were placed in homogenization tubes containing 1.4 mm ceramic beads. RNA was isolated by phenol–chloroform extraction using peqGold RNA TriFast (PeqLab, Germany). Therefore, samples were homogenized at 5.000 × g for 15 s. Total RNA amount and purity were determined with a Nanodrop 1000 photospectrometer (PeqLab, Germany) using A260/A280 as well as A260/230 ratios. cDNA was synthesized by reverse transcription using M-MLV reverse transcription (RT)-kit (Invitrogen, USA) and random hexanucleotide priming (Invitrogen, Germany). Gene expression levels were determined by real-time reverse transcription-PCR using SensiMix™ SYBR^®^ & Fluorescein Kit (Bioline, Germany) in a CFX connect Real-Time PCR system device (Bio-Rad, Germany). Primer sequences, individual annealing temperatures (AT), expected amplicon length are shown in Table 1. Results were evaluated using Bio-Rad CFX manager (Bio-Rad, Germany) and were normalized to a reference gene index (RGI), which was calculated from cyclophilin A and HSP90 expression data. Amplification efficiencies were calculated by standard curve analyses using a twofold dilution series of pooled samples. The target gene expression was calculated using efficiency corrected ΔΔCq method, using the geometric mean of both reference genes.

**Table 1:** List of used primers for PCR analysis, s: sense; as: anti-sense; AT: annealing temperature

Primer	Sequence [5′ → 3′]	AT [°C]	Amplicon length [bp]
HO-1	S: AAGCCGAGAATGCTGAGTTCA AS: GCCGTGTAGATATGGTACAAGGA	62	100
GCLC	S: GGGGTGACGAGGTGGAGTA AS: GTTGGGGTTTGTCCTCTCCC	65	125
Nqo1	S: CTACCCCCAGTGGTGATAGAAA AS: AGAGAGTGCTCGTAGCAGGAT	60	103
Txnrd1	S: GGGCTTCCACGTGCTGGGTC AS: TCCCCCAGAGCGCTTCGTCA	60	163
IL-6	S: GATACCACTCCCAACAGACCTG AS: GGTACTCCAGAAGACCAGAGGA	65	122
TNF-a	S: GCCATAGAACTGATGAGAGGGAG AS: GGTGCCTATGTCTCAGCCTCTT	62	139
CXCL10	S: CCAAGTGCTGCCGTCATTTTC AS: GGCTCGCAGGGATGATTTCAA	64	86
IL-1b	S: GCCCATCCTCTGTGACTCAT AS: AGGCCACAGGTATTTTGTCG	61	230
Cyclophilin A	S: TTGGGTCCAGGAATGGCAAGA AS: ACATTGCGAGCAGATGGGGT	64	148
Hsp90	S: TACTACTACTCGGCTTTCCCGT AS: TCGAATCTTGTCCAGGGCATC	64	191

### Protein biochemical analysis

Isolated tissues were mechanically disrupted in radioimmunoprecipitation assay (RIPA) buffer (pH 8.0) supplemented with a protease inhibitor cocktail (Complete Mini, Roche Diagnostics, Grenzach-Wyhlen, Germany). Protein concentrations were determined using the PierceTM BCA protein assay kit (Thermo Fisher Scientific, Waltham, USA) according to the manufacturer’s protocol. Same amounts of protein samples (approx. 20 μg per lane) were loaded, separated in a 12% SDS polyacrylamide gel by gel electrophoresis and transferred to a PVDF (polyvinylidene difluoride) membrane. After blocking with 5% skimmed milk in Tris-buffered saline containing 0.05% Tween 20 (TBS-T) for 1 h at room temperature, polyvinylidene difluoride (PVDF) membranes were incubated with primary antibodies (Table 2) overnight at 4 °C. An appropriate secondary antibody (1:5.000, see Table 2) was applied for 2 h at room temperature. Signals were analyzed via chemiluminescence detection (Westar Supernova, XLS 30100, Cyanagen, Italia), visualized (Fusion Solo X, Vilber, Germany) and subjected to densitometry analysis using Image J. Results were normalized to the expression of GAPDH or b-actin as reference protein.

**Table 2:** List of used antibodies

Antibody	Host species	Company	Co.No	Dilution factor		Secondary antibody, company
				IHC	WB	
HO-1	Mouse	Abcam	ab12348	–	1:200	Goat Anti-Mouse IgG, Sigma
4-HNE	Mouse	Abcam	ab48506	1:2000	–	Horse Anti-Mouse IgG, Vector
NQO1	Mouse	Thermo Fisher	39–3700	–	1:500	Goat Anti-Mouse IgG, Sigma
MBP	Rat	Abcam	ab7349	–	1:1000	Rabbit Anti-Rat IgG, abcam
Olig-2	Mouse	Millipore	MABN50	1:2000	–	Horse Anti-Mouse IgG, Vector
NeuN	Mouse	Millipore	MAB377	1:5000	–	Horse Anti-Mouse IgG, Vector
b-actin	Goat	Santa Cruz	sc1616	–	1:5000	Rabbit Anti-Goat IgG, Sigma
GAPDH	Rabbit	Santa Cruz	sc25778	–	1:5000	Goat Anti-Rabbit IgG, BIO-RAD
GFAP	Goat	Santa Cruz	sc6170	1:1000	1:1000	Horse Anti-Goat IgG, Voctor
S100b	Rabbit	Abcam	ab41548	1:10,000	–	Goat Anti-Rabbit IgG, Vector

### Immunohistochemistry and luxol fast blue staining

For immunohistochemistry, 5 µm thick sections of SC were deparaffinized, rehydrated, and antigens were unmasked by heating in Tris/EDTA (pH 9.0) buffer for 20 min. After blocking with 5% normal goat serum in PBS, the sections were incubated overnight at 4 °C with the primary antibody diluted in blocking solution. Primary antibodies and dilutions used in the study are given in Table 2. After washing and blocking of endogenous peroxidase with 0.3% hydrogen peroxide, slides were incubated with the appropriate secondary antibody (1:50; see Table 2) diluted in 5% normal serum in PBS for 1 h (RT). Afterwards, an incubation with ABC-solution (both parts 1:50, VECTASTAIN Elite ABC Kit (Standard), Vector Labs, PK-6100) diluted in PBS for 1 h (RT) followed. For visualization by 3′3-diaminobenzidine (DAB), the working solution (1:50, pH 7.5, DAKO, Hamburg, Germany) was applied for 10 min. Where appropriate, slides were counterstained with Mayer’s Haematoxylin (1 min) to visualize cell nuclei. The sections were washed again, dehydrated and mounted.

For Luxol Fast Blue (LFB) staining, sections were deparaffinized and hydrated, then incubated in a 0.1% LFB solution overnight at 56 °C. The next day, the excess of staining solution was rinsed off in 95% ethyl alcohol, washed in distilled water and differentiated in 0.05% lithium carbonate solution for 20 s. After this, periodic acid–Schiff (PAS) staining was performed by oxidizing the slides in 0.5% periodic acid solution for 5 min, rinsing in distilled water, and incubating in Schiff reagent for 15 min. Afterwards, sections were washed in tap water for 5 min. Slides were counterstained with Mayer’s hematoxylin for 1 min. The tissue was then washed again, dehydrated and mounted.

### Quantification of histological and immunohistochemical parameters

To evaluate demyelination in LFB stained sections, the magnitude of myelination was scored using image J software (ImageJ software, free Java software provided by the National Institute of Health, Bethesda, Maryland, USA). Quantification of Olig2, GFAP, Iba-1 and NeuN positive cells was performed by manually counting the number of positive signals in the epicenter of SC. For each animal, a total of four slices were analyzed with a distance of 100 μm in between. Pictures were taken using a LEICA DFC365 microscope and cell numbers are expressed as positive cells per mm^2^. All morphological quantification was performed in a blinded way, where the observer was not aware of the treatment.

### Statistical analysis

GraphPad Prism 8 (GraphPad Software Inc., San Diego, CA, USA) was used for statistical analyses. Brown–Forsythe test was performed to test for equal variances and normal distribution was tested with Shapiro–Wilk test. If necessary, data were transformed via Box–Cox-Y to achieve homoscedasticity. One-way ANOVA followed by Dunnett post-hoc test or two-way ANOVA followed by Tukey post-hoc test (in case of significant interaction of parameters) was used for parametric data. Non-parametric data were analyzed with Kruskal–Wallis test followed by Dunn’s multiple comparisons or Friedman test. All data are given as arithmetic means ± standard deviation (SD). *p* values < 0.05 were considered significant. Detailed description of significance levels are given in the figure legends.

## Results

### Activation of the Nrf2/ARE pathway after SCI

**Fig. 1:**
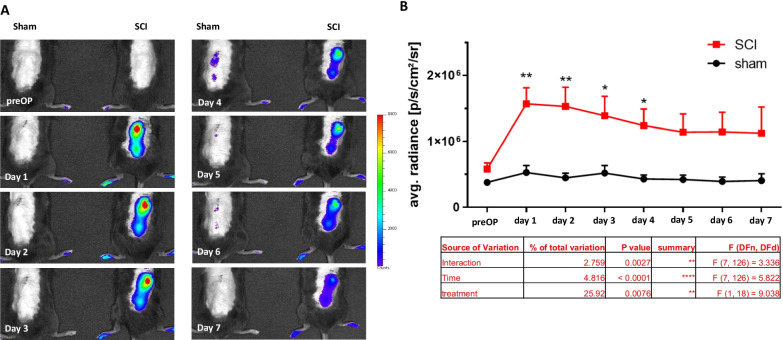
Longitudinal activation pattern of the Nrf2/ARE system in the SCI model. **A** Shows representative pictures of the luminescence measurements of each time point. **B** Quantification of luminescence intensity, depicted as average radiance [photons/second/cm^2^/steradiant], during the first 7 days post SCI. *p < 0.05; **p < 0.01 vs. same time point of sham. Data represent means + SD, *n* = 6

**Fig. 2:**
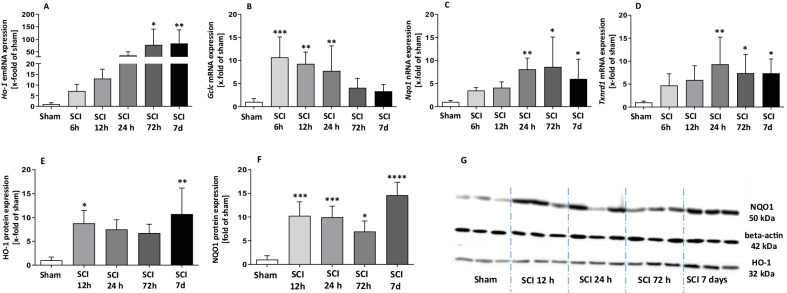
Measurement of Nrf2 target genes. **A**–**D** Gene expression was investigated using RT-qPCR: HO-1 (heme oxygenase-1), GCLC (glutamate–cysteine ligase, catalytic subunit), NQO1 (NADPH quinone oxidoreductase 1) and TxnRD1 (thioredoxin reductase 1). **E**–**G** Show the protein levels of HO-1 and NQO1 at 12, 24, 72 h and 7 day post SCI. *p < 0.05; **p < 0.01, ***p < 0.001 vs. sham. Data represent means + SEM, qPCR: *n* = 5; WB *n* = 3

To investigate whether the Nrf2/ARE system plays a role in our experimental SCI animal model and to assess the quantitative pattern of its activation, we included transgenic ARE-Luciferase reporter gene mice. In this approach, due to the ARE-driven expression of the luciferase, the luminescence intensity directly correlates with Nrf2-activity. Using the XENOGEN Living Image system, the luminescence signals in SCI and sham mice were daily measured in vivo. As shown in Fig. 1, compared to sham control animals a significant increase of luciferase activity was observed in the epicenter of SC in SCI mice as early as 24 h post injury (1.57 × 10^6^ ± 2.4 × 10^5^ p/s/cm^2^/sr vs. 5.16 × 10^5^ ± 1.24 × 10^5^ p/s/cm^2^/sr). The luminescence intensity gradually declined during the study but remained significantly higher for up to 4 days compared to sham controls (Fig. 1A, B). Afterwards, the mean signal intensities of SCI mice were still higher than those of sham control animals but without reaching statistical significance. These experiments indicate an involvement of Nrf2-activity in our contusion model of SCI. Furthermore, to confirm in vivo bioluminescence analysis, we measured the expression of four typical Nrf2-target genes including heme oxygenase-1 (HO-1), glutamate–cysteine ligase catalytic subunit (GCLC), NAD(P)H dehydrogenase [quinone] 1(NQO1) and thioredoxin reductase-1(TXNRD1) by RT-qPCR. These results showed an induction of the gene expression of all selected genes (Ho-1 after 72 h: *F*(5, 26) = 6.113, 77.20 ± 63.45 fold of sham; Gclc after 6 h: *F*(5, 26) = 7.669, 10.63 ± 4.44 fold of sham; Nqo1 after 24 h: 8.06 ± 2.46 fold of sham; Txnrd1 after 24 h: *F*(5, 26) = 3.741, 9.31 ± 5.96 fold of sham) in epicenter after SCI (Fig. 2A–D). To further investigate this in more detail, we analyzed protein levels of HO-1 and NQO1 in SC using WB. As shown in Fig. 2E–G, protein expression of HO-1 and NQO1 was significantly induced as early as 12 h post-OP (HO-1: *F*(4, 10) = 4.439, 8.76 ± 2.68 fold vs. 1.00 ± 0.71 fold of sham; NQO1: *F*(4, 14) = 14.81, 10.24 ± 2.95 fold vs. 1.00 ± 0.84 fold of sham) and remained at comparable levels until day 7. These results indicate the critical role of the Nrf2-regulated anti-oxidant machinery in the early phase after SCI.

### Nrf2 hyperactivation improves the functional recovery

**Fig. 3:**
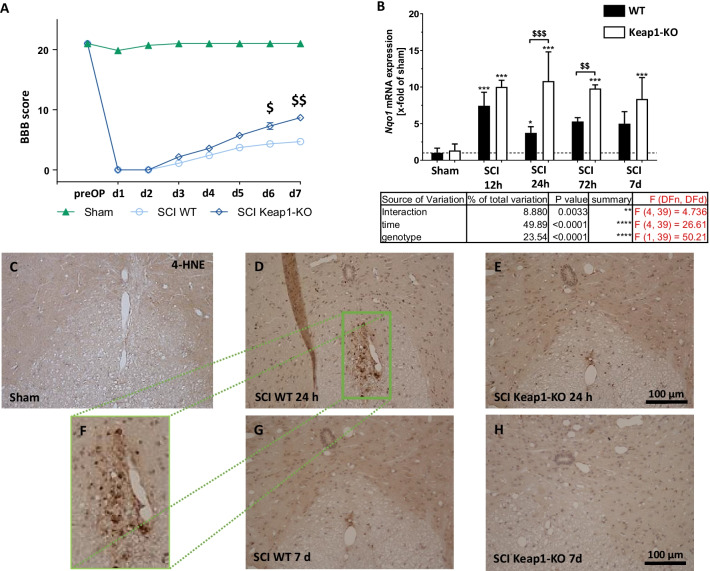
Locomotive outcome and oxidative stress after SCI. **A** BBB locomotion test was used to assess the motor function in hind-limbs during the first 7 day after SCI. There was no significant difference from days 1 to 5 between WT and knockout mice. On days 6 and 7, we observed a significantly improved functional recovery in Keap1-KO mice. **B** Shows the gene expression of the Nrf2 target gene Nqo1, in both mice strains and at all investigated time points. Sham vs. SCI WT/SCI Keap1-KO *p < 0.05. Sham vs. SCI WT/SCI Keap1-KO ***p < 0.001. SCI WT vs. SCI Keap1-KO ^$^*p* < 0.05; SCI WT vs. SCI Keap1-KO ^$$^*p* < 0.01. SCI WT vs. SCI Keap1-KO ^$$$^*p* < 0.001. Data represent means + SD. **C**–**H** Immunhistochemical detection of oxidative stress related damage by the means of 4-Hydroxy-nonenal (4-HNE) after 24 h (C–E) and 7 days in both genotypes (**F** and **G**) post SCI. BBB: *n* = 8; qPCR: *n* = 4

In the next step, to investigate the effect of astrocytic Nrf2-activation on locomotor recovery after SCI, we performed the BBB scoring for 7 consecutive days after injury. During the first 5 d after insult, there was no significant difference between WT and Keap1-KO mice (Fig. 3A) even if the mean score of Keap1-KO was slightly higher than the score of WT mice. At days 6 and 7 post injury, the Keap1-KO group showed a significantly higher BBB scoring compared to WT animals which indicates that increased Nrf2 activity improves the recovery of locomotor abilities in SCI mice. In a further attempt, we explored whether Nrf2-target genes are regulated in Keap1-KO mice. The RT-qPCR data revealed that mRNA expression levels of Nqo1 in Keap1-KO mice were significantly higher than in WT mice at 24 and 72 h post SCI (Fig. 3B, WT vs. Keap1-KO; 24 h: 3.74 ± 0.85 fold vs. 10.81 ± 4.00 fold of sham; 72 h: 5.29 ± 0.53 fold vs. 9.80 ± 0.49 fold of sham), whereas the expression level of Ho-1 showed no significant changes in both mice strains (data are not shown). Furthermore, we compared the distribution and immunoreactivities of 4-hydroxy-2E-nonenal (4-HNE) a major cytotoxic end product of lipid peroxidation, in epicenter of SCI. As shown in Fig. 3C–H, 4-HNE-immunoreactive cells were well observed in WT mice at 24 h post SCI (Fig. 3D).

### Sustained elevated Nrf2/ARE pathway protects spinal cord tissue

We have shown that the activation of the Nrf2/ARE pathway in the epicenter of the SC is a characteristic feature of our SCI model. We, therefore, aimed at investigating whether a continuous hyperactivation of Nrf2 might prevent cellular pathological responses in traumatic SCI. Since astrocytes are critically involved in the maintaining of hemostasis during SCI, Keap1 was deleted specifically in this cell type, representing a genetic model for sustained activation of the Nrf2/ARE pathway in astrocytes [29].

**Fig. 4:**
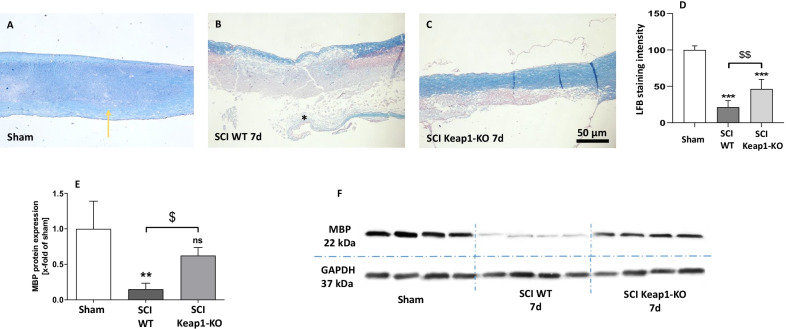
Analyses on myelination and involved proteins. **A**–**D** Myelin staining intensity (LFB) was markedly reduced in SC post SCI. Nrf2 hyperactivation protected SC from demyelination post SCI in epicentral (**A**–**D**). **E** and **F** Using western blotting, the amount of myelin basic protein (MBP) (main protein for myelin assembly) was measured in WT and Keap1-KO mice. The myelination index of LFB-stained sections was significantly restored in knockout mice compared to WT animals. Scale bars: 50 µm. sham vs. SCI WT/SCI Keap1-KO *p < 0.05. Sham vs. SCI WT/SCI Keap1-KO **p < 0.01; sham vs. SCI WT/SCI Keap1-KO ***p < 0.001; SCI WT vs. SCI Keap1-KO ^$^*p* < 0.05. SCI WT vs. SCI Keap1-KO ^$$^*p* < 0.01. Data represent means ± SD; LFB: *n* = 6; WB *n* = 4

To demonstrate its effect on the prevention of demyelination, sagittal sections of SC were stained with LFB. In sham operated mice, LFB staining of myelin in the SC was mainly restricted to the white matter (yellow arrows Fig. 4A). The microscopic observations revealed that at 7 days post SCI, LFB staining was strongly reduced with almost no staining visible in the dorsal column at the epicenter (Fig. 4B, central: 21.47 ± 8.90% LFB intensity of ham) of the injury (stars in Fig. 4B). In addition, most of the ventral column also showed a partially reduction of staining in the epicenter. In sharp contrast, the loss of myelin was significantly prevented in Keap1-KO mice (Fig. 4D, central: 46.30 ± 13.24% LFB intensity of sham) of SC (Fig. 4C).In addition, we observed the same protective effect in caudal part of injury in Keap1-KO mice (Additional file 1: Fig. S2A–D).

Since myelin basic protein (MBP) is one of the main proteins known to be required for myelin assembly, we measured the amount of this protein in WT and Keap1-KO mice. Our results show [*F*(2, 8) = 12.65] that SCI significant reduced MBP in WT mice (0.15 ± 0.09-fold of sham) and that this effect is ameliorated in Keap1-KO animals (0.63 ± 011-fold of sham), which also showed a decreased amount of MBP but significantly more than WT mice (Fig. 4E, F).

**Fig. 5:**
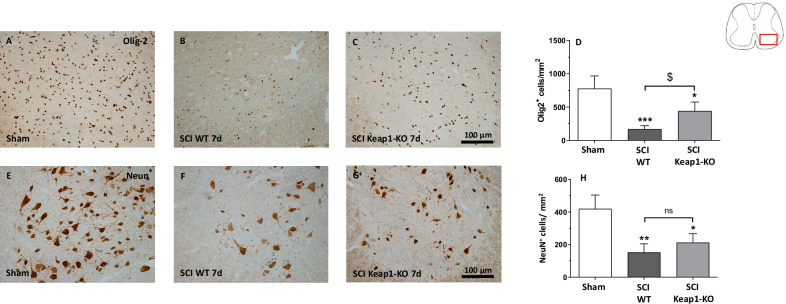
Effect of hyperactivated Nrf2 in astrocytes on oligodendrocytes and neuronal cell numbers post SCI. **A**–**C** Representative microphotographs of Olig2-positive cells of different experimental groups. **D** Quantification of Olig2-positive cells/mm^2^. The amount of Olig-2-positive is reduced in both genotypes after SCI, but with significantly higher number of remaining Olig2-positive cells in Keap1-KO mice. **E**–**G** Representative microphotographs of NeuN positive cells (neuronal marker) of different experimental groups. **H** Quantification of NeuN-positive neurons in the ventral horn of the spinal cord is presented in different groups. Scale bars: 100 µm. SCI WT/SCI Keap1-KO *p < 0.05. Sham vs. SCI WT/SCI Keap1-KO **p < 0.01; sham vs. SCI WT/SCI Keap1-KO ***p < 0.001.; SCI WT vs. SCI Keap1-KO ^$^*p* < 0.05. Data represent means ± SD; *n* = 4

To further examine whether the suppression of demyelination is correlated with improved survival of oligodendrocytes in Keap1-KO mice, we conducted immunohistochemical detection with anti-Olig2 antibody. The quantifications [treatment *F*(2, 9) = 19.78] of Olig2 immunoreactive showed that SCI induced a significant loss of Olig2 positive cells in the gray and white matter (Fig. 5B). While still reduced compared to sham-operated mice (777.6 ± 190.0 Olig2^+^ cells/mm^2^), we found significantly higher numbers of Olig2-positive cells in Keap1-KO animals (442.4 ± 133.7 Olig2^+^ cells/mm^2^) compared to WT mice (167.6 ± 51.54 Olig2^+^ cells/mm^2^) after SCI (Fig. 5A–D). To investigate whether increased astrocytic Nrf2-activity protected neurons against SCI-mediated damage, neuronal density was evaluated using immunohistochemistry against NeuN (a specific neuronal protein). We measured a significant decline of NeuN-positive cells [treatment *F*(2, 9) = 18.56] in WT mice (152.50 ± 52.05 NeuN^+^ cells/mm^2^) after SCI and a similar reduction in Keap1-Ko mice (212.60 ± 53.92 NeuN^+^ cells/mm^2^) compared to sham animals (420.00 ± 84.35 NeuN^+^ cells/mm^2^) (Fig. 5E–H).

### Increased astrocytic Nrf2 activity ameliorates neuroinflammation after SCI

**Fig. 6:**
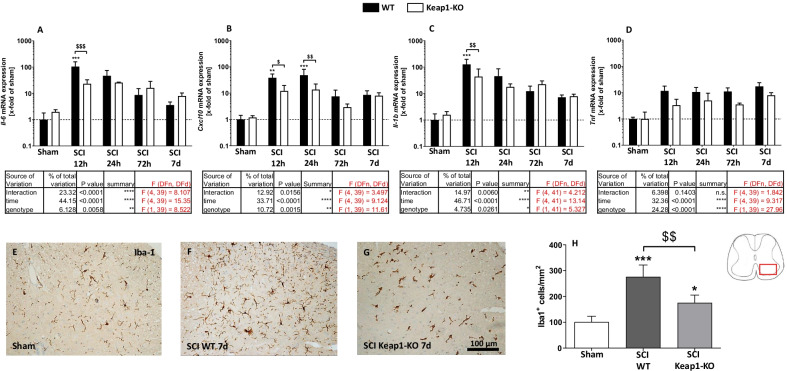
Analyses on neuroinflammatory processes in the course of SCI. **A**–**D** Regulation of gene expression of different pro-inflammatory cytokines in both WT and Keap1-KO mice at different time points. All measured cytokines, [interleukin 6 (IL-6), C-X-C motif chemokine ligand-10 (CXCL10), interleukin-1b (IL-1b) and tumor necrosis factor alpha (TNF-α)], significantly increased in the early phase post SCI. This effect is partially suppressed in Keap1-KO mice. **E**–**G** Show the quantitative evaluation of microglia/macrophages (Iba-1) cell numbers in SC 7 days after SCI and representative microphotographs of the different experimental groups. Note that microglia/macrophage responses are significantly reduced in Keap1-KO mice. Scale bars: 100 µm. Sham vs. SCI WT/SCI Keap1-KO *p < 0.05. Sham vs. SCI WT/SCI Keap1-KO **p < 0.01; sham vs. SCI WT/SCI Keap1-KO ***p < 0.001; SCI WT vs. SCI Keap1-KO ^$^*p* < 0.05; SCI WT vs. SCI Keap1-KO ^$$^*p* < 0.01. Data represent means ± SD; qPCR: *n* = 4–6; IHC: *n* = 4

To determine the influence of astrocytic Nrf2 activation on neuroinflammation, mRNA expression of pro-inflammatory cytokines (IL-6, CXCL10, IL-1b and TNF-α) were analyzed in WT and Keap1-KO mice at various time points (12 h, 24 h, 72 h and 7 days) after SCI. The analyses revealed that IL-6, CXCL10 and IL-1b mRNA expressions immediately rose within the first 12 h post injury and then gradually declined up to 7 days post SCI (Fig. 6A–C). However, these inflammatory factors were significantly lower at the early phase of injury (12 h) in the Keap1-KO mice (Il-6: 23.29 ± 9.72-fold; Cxcl10: 12.12 ± 7.37-fold and Il-1b: 43.82 ± 41.35-fold of sham) in comparison to WT (Il-6: 104.63 ± 55.07-fold; Cxcl10: 38.44 ± 15.35-fold and Il-1b: 126.93 ± 69.89-fold of sham) animals (Fig. 6A–C). Moreover, TNF-α was up-regulated in the early phase of SCI and remained constantly at this high level until 7 days after SCI. The expression of this cytokine showed a significant genotype effect with lower mean values in knock-out mice (Fig. 6D). Furthermore, Iba-1 staining revealed a strong microgliosis [treatment *F*(2, 9) = 26.38] in both SCI-treated animals compared to sham (101.20 ± 22.37 Iba1^+^ cells/mm^2^). However, this pathological feature was significantly attenuated in Keap1-KO mice (175.60 ± 30.05 vs. 276.30 ± 45.90 Iba^+^ cells/mm^2^ in SCI-treated WT mice) (Fig. 6E–H).

### Astrocyte reactivity in Keap1-KO mice

**Fig. 7:**
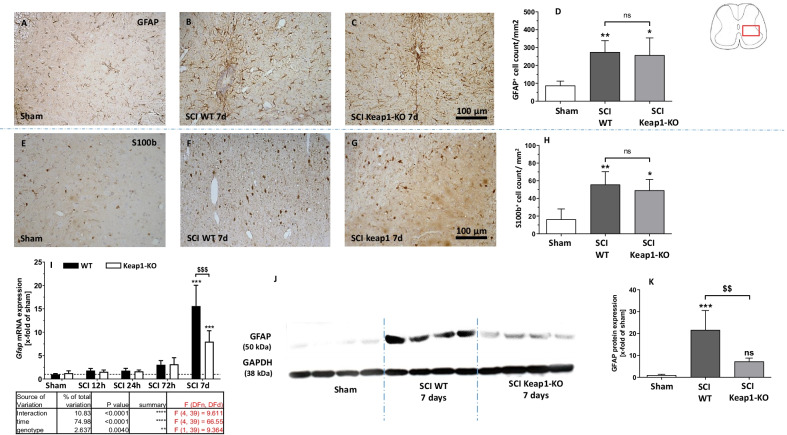
Analyses on astrogliosis and astrocyte reactivity. **A**–**H** Assessment of number of astrocytes 7 day post SCI using two different markers, GFAP and S100b. **I** Gene regulation of GFAP in WT and Keap1-KO at different time points. **J**, **K** Show the protein levels of GFAP in both WT and Keap1-KO mice 7 day post SCI. Scale bars: 100 µm. Sham vs. SCI WT/SCI Keap1-KO *p < 0.05. Sham vs. SCI WT/SCI Keap1-KO **p < 0.01; sham vs. SCI WT/SCI Keap1-KO ***p < 0.001; SCI WT vs. SCI Keap1-KO ^$$^*p* < 0.01. SCI WT vs. SCI Keap1-KO ^$$$^*p* < 0.001. Data represent means ± SD; IHC: *n* = 4; qPCR: *n* = 4–6; WB: *n* = 4

A main characteristic feature of SCI is the accumulation of reactive astrocytes identified as astrogliosis. Therefore, our next approach was to investigate the activation of astrocytes in WT as well as Keap1-KO mice after SCI. As expected, SCI in WT mice increased the number of GFAP- (Fig. 7B, D; 274.60 ± 64.63 cells/mm^2^) and S100b- (Fig. 7F, H; 55.76 ± 14.56 cells/mm^2^) positive cells in the SC compared to sham treated animals (Fig. 7A, E; 87.45 ± 25.36 GFAP^+^ and 16.38 ± 11.75 S100b^+^ cells/mm^2^, respectively). This induction in epicenter was comparable in both mouse strains and was not abolished in Keap1-KO mice (Fig. 7C, D and G, H; 257.7 ± 96.85 GFAP^+^ and 49.20 ± 12.27 S100b^+^ cells/mm^2^, respectively). In addition, mRNA and protein levels of GFAP were examined using RT-qPCR and WB, respectively. Although the number of GFAP-positive cells did not differ between genotypes, we observed a significant reduction of GFAP after 7 days at both the mRNA (Fig. 7I) as well as the protein (Fig. 7J, K) level in Keap1-KO mice compared to WT animals (mRNA: 7.97 ± 2.35-fold vs. 15.57 ± 4.46-fold of sham; protein: 7.21 ± 1.55-fold vs. 21.60 ± 8.87-fold of sham). This indicates that Nrf2 may rather affect the activation/reactivity of astrocytes than their proliferation.

## Discussion

Spinal cord injury (SCI) has been shown to be associated with different kind of tissue damage [30]. Besides others, oxidative stress has frequently been described to be involved during SCI pathogenesis [31]. While oxidative eustress (low oxidant exposure) regulates a couple of intracellular redox-regulated processes to maintain redox-homeostasis [6], oxidative distress represents an excess of oxidants leading to tissue damage. The nuclear factor erythroid-2 related factor 2 (Nrf2) regulates a battery of genes that encode for anti-oxidative enzymes that are part of eustress-related signaling. We demonstrated that the Nrf2/ARE system was activated immediately in the course of SCI-induced redox signaling. However, significant tissue damage occurs in this model despite Nrf2 activation, suggesting that the physiological Nrf2 response is insufficient to adequately counteract the full extent of this damage [32].

Fu and colleagues just recently demonstrated that luteolin pre-treatment, a flavonoid known to activate Nrf2 and to exert antioxidant properties, improved SCI-related burden in rats [33]. Besides from luteolin, there have been other Nrf2 activating substances used in similar studies [34–36]. However, all these studies share a common drawback that these compounds have a variety of other intracellular effects in addition to Nrf2 activation, which are not always completely known.

Astrocytes are prominent in the cellular response to SCI which maintain the neuronal structure and nutrition. Furthermore, it has been shown that astrocytes provide protection against different kind of damage, including oxidative stress [37]. For this reason, a more detailed study of the role of Nrf2 specifically in astrocytes during SCI, which has not been addressed in previous studies, would be of scientific value.

Therefore, in addition to wildtype mice we used transgenic GFAP-mediated Keap1-KO mice to establish a sustained Nrf2 activation in astrocytes. In contrast to before-mentioned studies, in which the treatment regiments induce a systemic Nrf2 activation, we were able to show that Nrf2 hyperactivation exclusively in astrocytes was absolutely sufficient to overcome SCI-related outcomes. In particular, our study revealed that permanent Nrf2 activity not only protected against general tissue damage (including demyelination and loss of olig-2 positive cells), but also prevented the intracellular redox milieu from switching to oxidative distress.

Neuroinflammatory response is one of the main events which occurs within early hours from the onset of SCI, which contributes to neuronal death and further neurologic damages. The neuroinflammation is mainly characterized by the infiltration of inflammatory cells, such as macrophages as well as the accumulation of inflammatory mediators like IL-1b and IL-6. In this study we observed that Keap1-KO mice showed a significantly lower expression of pro-inflammatory cytokines and also SCI-induced microgliosis, suggesting that increased astrocytic activity of Nrf2 plays a critical role in counteracting the inflammation. Even though microgliosis was clearly reduced in Keap1-KO mice, astrocyte numbers did not alter. However, in the context of neuroinflammation not simply the amount of astrocytes but rather their reactivity is crucial for tissue damage [38]. Because the expression of GFAP, one of the prime markers of astrocyte reactivity, was significantly reduced in Keap1-KO mice, it is reasonable to conclude that Keap1 depletion significantly reduces the astrocyte-triggered inflammatory response, despite unchanged astrocyte numbers. This is consistent with the results of one of our previous studies showing that astrocytic Keap1-KO reduced neuroinflammation and tissue damage in a mouse model of multiple sclerosis, although the number of astrocytes also did not differ from treated WT mice [27]. Several evidences confirm that Nrf2 can limit the inflammatory response by inhibiting NF-kB activation through maintenance of redox homeostasis [39, 40]. This important protective function of Nrf2 is mainly mediated through its antioxidant and detoxifying enzymes, such as NQO1. Thus, enhancement of these antioxidant systems, as is the case in astrocytes of Keap1-KO mice, could lead to decreased production of pro-inflammatory cytokines. This is particularly also important, because, among other functions, astrocytes are responsible for maintaining a suitable environment for neurons including redox homeostasis. To ensure this, they contribute significantly to the reduction of oxidative distress by counteracting harmful concentrations of reactive oxygen species (ROS) in their surrounding area [41]. Since increased emergence of ROS not only leads to oxidative damage of biomolecules but also activates microglial cells, astrocytes with increased antioxidant defenses may exert a direct inhibitory influence on microgliosis [41]. However, not only astrocytes, but microglia as well as pericytes play decisive roles during SCI-induced neuroinflammation and subsequent recovery. While microglia directly influence the neuroinflammatory response, pericytes have an indirect influence on inflammation and regeneration after SCI due to their roles as a functional component of the neurovascular unit, such as the development and maintenance of the blood–spinal cord barrier [42]. In addition, pericytes also appear to be involved in scar formation after SCI [43], both crucial processes in the course of traumatic spinal cord injury. It has already been shown that Nrf2 has a major influence on the inflammatory response of various immune cells, such as macrophages [44–46] to which also microglial cells belong. The literature provides several lines of evidence that microglial Nrf2 reduces the inflammatory reactivity of these cells, by inhibiting NF-kB [47] among other factors, and drives the cells into a more anti-inflammatory phenotype [47]. With regard to pericytes, it has not yet been examined whether and what influence intrinsic Nrf2 might have on the functions of these particular cells during the course of SCI. Unfortunately, such research questions were not addressable with our current genetic approach, but it would be particularly interesting to investigate in upcoming studies.

Hind limb gait analysis was performed with BBB score which is a monotonic scoring method based on observation of locomotor behavior in the recovery phase after SCI. The original BBB model is suitable for rat, but recently in many studies, the BBB scale was also applied to the assessment of functional outcomes in spinal cord injured mice [48–51]. Other established locomotor tests for spinal cord injured animals, such as the “Tarlov scale” [52] and the inclined-plane test [49], would not be appropriate for use in SCI-treated mice due to the difficulties in obtaining precise measurements in restless mice. Since mouse recovery differed from rats for coordination, paw position and trunk instability and also due to their rapid movement and smaller size, in our study we have used the modified version of test for mice which was designed by Joshi and Fehlings [50]. Using BBB scoring, we are able to consider an accurate method to track motor function recovery following SCI. The BBB scale scores should be assessed by multiple blind examiners, because differences in scores among examiners are often seen. However, inexperienced observers are able to quickly learn the scoring method . Although the BBB scale correlates with the exploratory activity of animals with low motor ability; in the higher range (≥ 13 points), this scale includes rather discrete aspects of movement that do not represent major improvements in an animal’s ability, and the sequence of recovery is often not related to the scale ranking [48]. However, in this study, since we evaluated the BBB score only in the early phase of SCI (up to 7 days), this detrimental factor did not influence our analysis. The other more sensitive method to assess the locomotion rating scale in mice is Basso Mouse Scale for Locomotion (BMS). BMS is more highly susceptible to small biomechanical gradations which designed by Basso et al. [53] for ideosyncratic recovery of various mouse strains. In general, the BBB score is principally based on three specific motion parameters: (1) movements of the joints in isolated positions; (2) frequencies of coordination and (3) clearance of the toes [54]. As mentioned above, the small body size and high locomotion speed of mice led to a distinct range of variation in the evaluation of these parameters. Therefore, in the BMS, these rankings were modified or eliminated from the BBB scale. One of the major alterations refers to the assessment of isolated joint movements. Meanwhile, it was evident that even skilled observer had challenges in recognizing whether the hip and knee joint movements during the forward progression. In mice, the movement of these joints was particularly slight and easily obscured by skin and hair folds. In contrast, ankle movements were quite distinct and the extent of ankle mobility could be easily assessed. Hence, only the ankle movements are assessed in BMS scale.

Since this study is conducted in male mice, our findings are only relevant for the male gender. Many evidences are emerging that the CNS responds differently to injury in males and females, and that gender-specific differences in pathophysiological processes exist. There may be a number of possible mechanisms regarding the observed difference in magnitude of both the initial injury and recovery thereafter. One of these might depend on the neuroprotective and/or neuroregenerative actions of estrogen and the detrimental effects of testosterone [55]. Results of the small number of published studies comparing recovery among genders after SCI have been controversial. For example comparative examination of the extent of tissue damage or loss following experimental SCI has revealed in these studies that female animals exhibit significantly less tissue loss than males [56, 57]. However, clinical studies have suggested that a similar gender biased recovery may exist in human SCI [58–60]. Therefore, well-controlled studies with appropriate ovariectomized and castrated control groups, and covariant analysis for weight and age are needed to replicate these findings.

## Conclusions

In summary, our in vivo bioluminescence data showed Nrf2–ARE activation during primary phase of SCI. Furthermore, we were able to demonstrate that hyperactivation of Nrf2 improves functional recovery, prevents the neuroinflammation and demyelinating. Better understanding of underlying mechanisms of oxidative stress could help us to promote new therapeutic intervention in SCI patients.

## Supplementary Information

**Additional file 1: Figure S1**. (A) Schematic representation of different parts of spinal cord (rostral, epicenter and caudal); (B) measurement ARE-activity by correcting luminescence signals from pre-defined region of interest (ROIs) using background ROI. **Figure S2.** (A–D) Myelin staining intensity (LFB) in caudal part of injury and in different experimental groups. **Figure S3.** Images of the blots of Fig. 4.

## Data Availability

The data sets analysed during the current study are available from the corresponding author on reasonable request.

## Abbreviations

4-HNE: 4-Hydroxynonenal, or trans-4-hydroxy-2-nonenal
ARE: Antioxidant response element
b-actin: Beta-actin
BBB: Basso, Beattie, and Bresnahan score
BMS: Basso Mouse Scale
CNS: Central nervous system
CXCL10: C-X-C motif chemokine 10
DAB: 3′3-Diaminobenzidine
DMF: Dimethyl fumarate
EDTA: Ethylenediaminetetraacetic acid
GAPDH: Glyceraldehyde 3-phosphate dehydrogenase
GFAP: Glial fibrillary acidic protein
Ho-1: Heme oxygenase-1
Hsp90: Heat shock protein 90
Iba-1: Microglial marker, also called allograft inflammatory factor 1
IL-1b: Interleukin-1 beta
IL-6: Interleukin-6
Keap1: Kelch-like ECH-associated protein 1
KO: Knockout
LFB: Luxol fast blue
MBP: Myelin basic protein
NeuN: Neuronal marker, also called hexaribonucleotide binding protein-3
NF-kB: Nuclear factor ‘kappa-light-chain-enhancer’ of activated B-cells
Nqo1: NAD(P)H dehydrogenase [quinone] 1
Nrf2: Nuclear factor erythroid 2-related factor 2
Olig-2: Oligodendrocyte transcription factor 2
OS: Oxidative stress
PAS: Periodic acid–Schiff
PBS: Phosphate-buffered saline
PFA: Paraformaldehyde
RGI: Reference gene index
RIPA: Radioimmunoprecipitation assay
ROI: Region of interest
ROS: Reactive oxidant species
RT: Room temperature
S100b: S100 calcium-binding protein B
SC: Spinal cord
SCI: Spinal cord injury
TBS-T: Tris-buffered saline containing 0.05% Tween 20
TNF-a: Tumor necrosis factor alpha
Txnrd1: Thioredoxin reductase-1
WB: Western blot
