# Supplementary figures and images for: Astrocytic Nrf2 expression protects spinal cord from oxidative stress following spinal cord injury in a male mouse model
Source: J Neuroinflammation. 2022 Jun 6;19:134. doi: 10.1186/s12974-022-02491-1 (PMC9169394; doi:10.1186/s12974-022-02491-1)

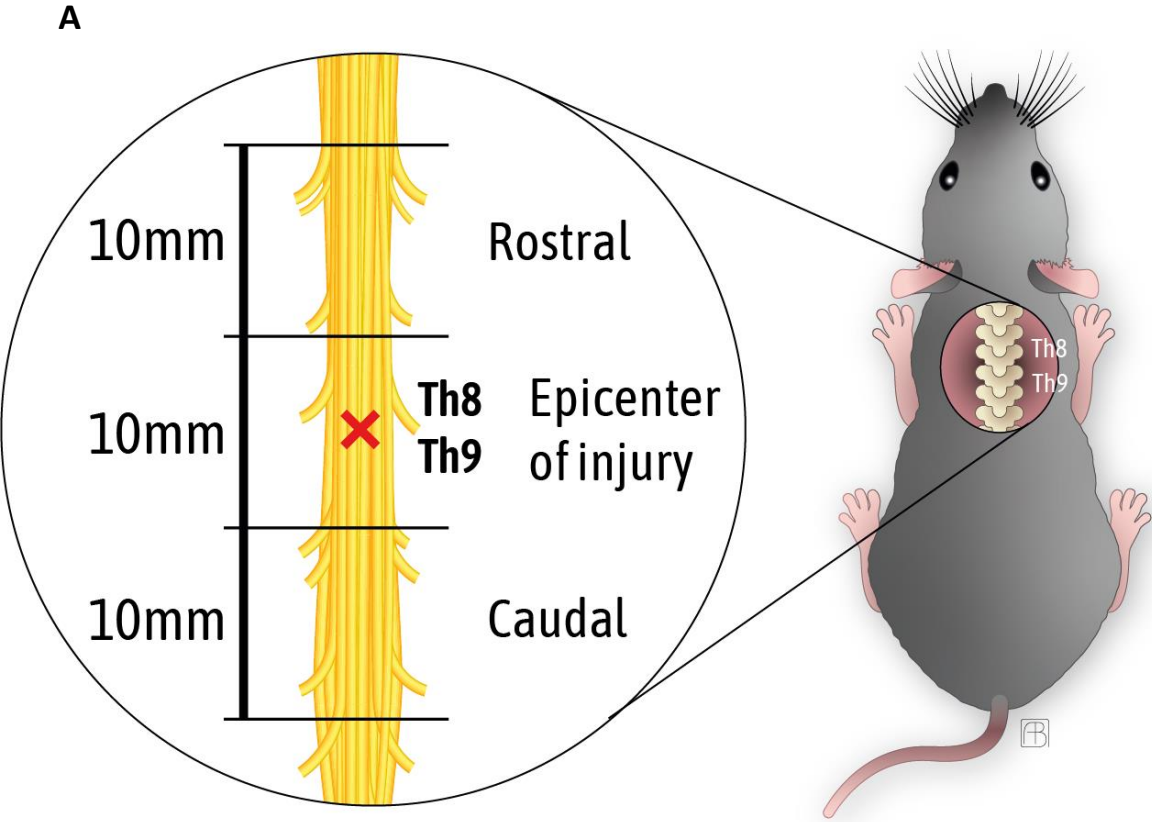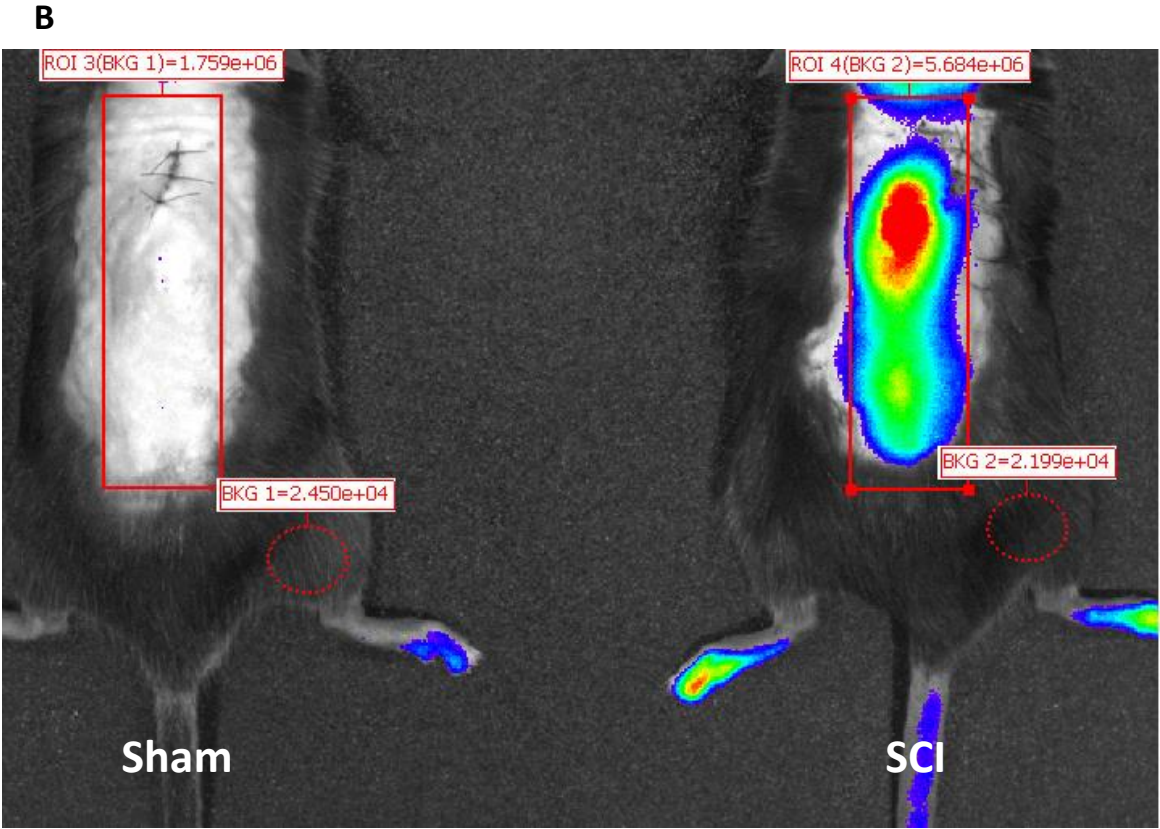

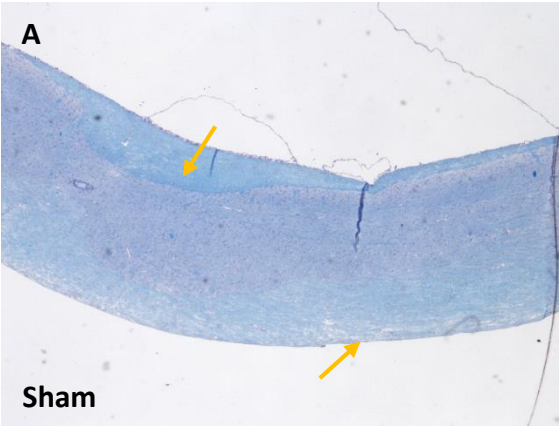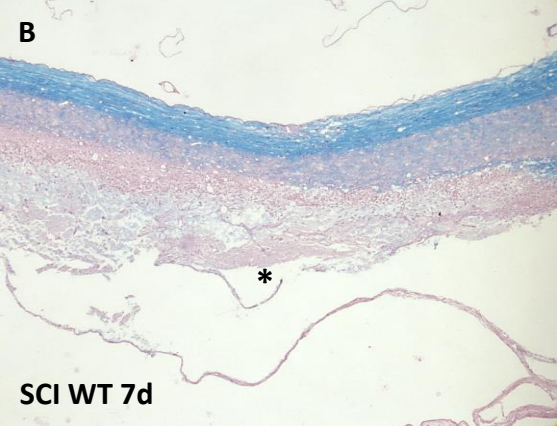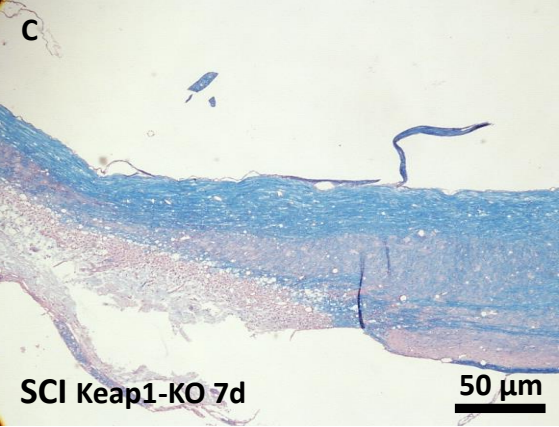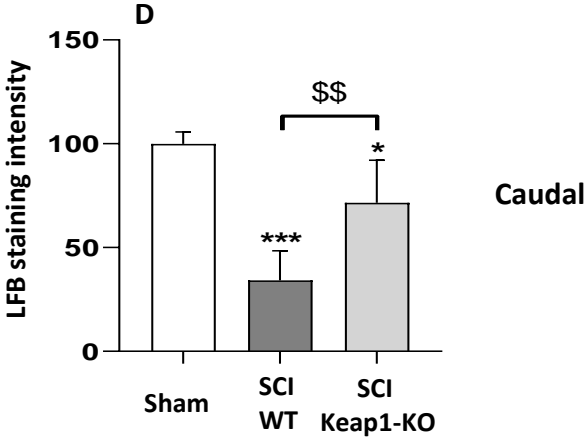

NQO1  
50 kDa

beta-actin  
42 kDa

HO-1  
32 kDa

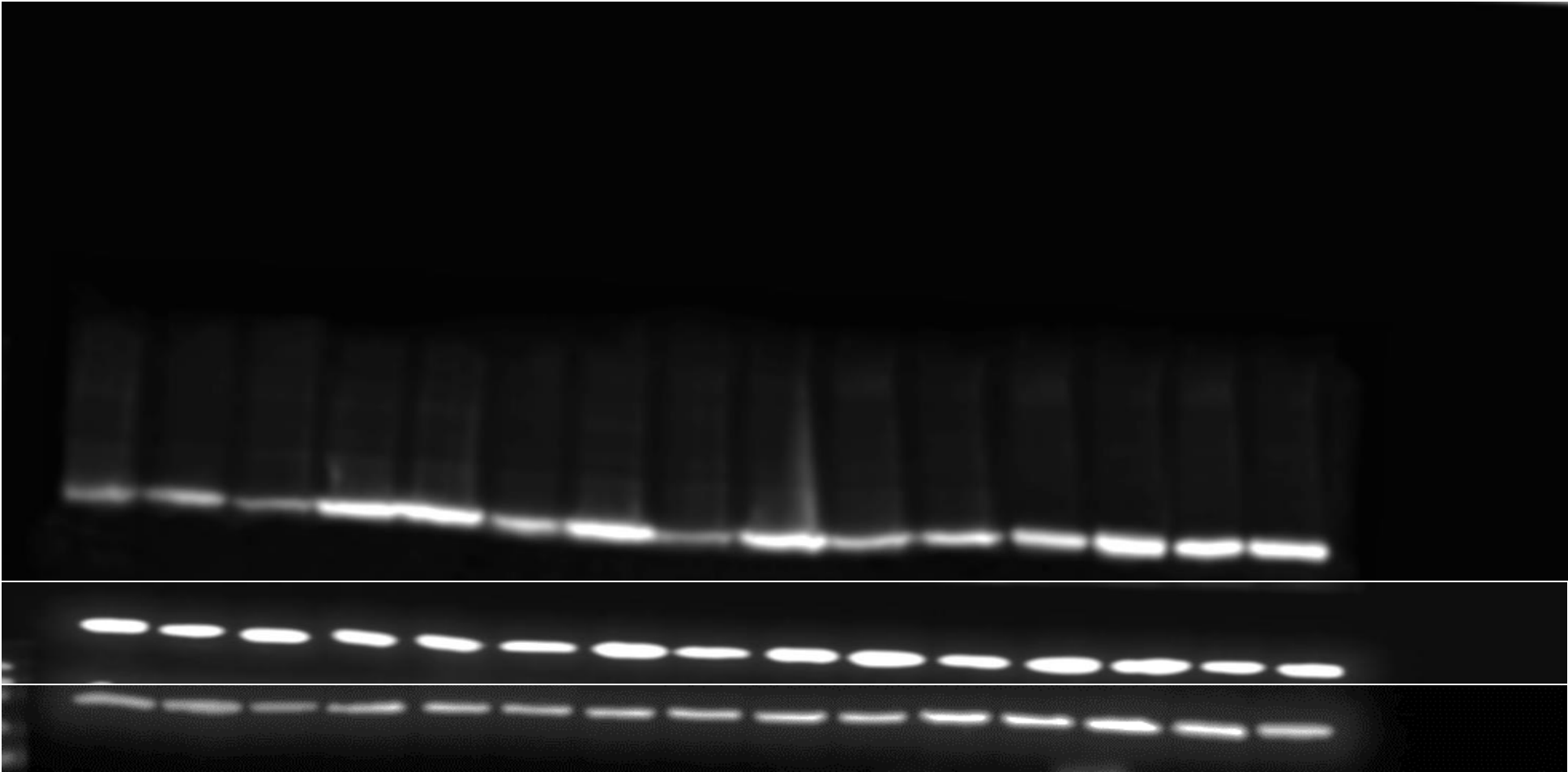

Supplement: Supplementary file 1 — Additional file 1: Figure S1. (A) Schematic representation of different parts of spinal cord (rostral, epicenter and caudal); (B) measurement ARE-activity by correcting luminescence signals from pre-defined region of interest (ROIs) using background ROI. Figure S2. (A–D) Myelin staining intensity (LFB) in caudal part of injury and in different experimental groups. Figure S3. Images of the blots of Fig. 4. [file 12974_2022_2491_MOESM1_ESM.pdf]
